# Yellow enhance mode is useful for distinguishing tissues in endoscopic transnasal surgery: case series with preliminary results

**DOI:** 10.1007/s10143-025-03485-2

**Published:** 2025-04-02

**Authors:** Hirotaka Hasegawa, Yuki Shinya, Motoyuki Umekawa, Satoshi Koizumi, Yoshiaki Goto, Satoshi Kiyofuji, Shunya Hanakita, Masahiro Shin, Masao Iwagami, Nobuhito Saito

**Affiliations:** 1 Department of Neurosurgery, The University of Tokyo, Bunkyo, Tokyo Japan; 2 Department of Neurosurgery, Saitama Medical Center, Saitama Medical University, Saitama, Japan; 3 Department of Neurosurgery, Teikyo University, Itabashi, Tokyo Japan; 4 Department of Digital Health, Institute of Medicine, University of Tsukuba, Tsukuba, Ibaraki Japan

**Keywords:** Endoscopic transnasal surgery, Image-enhanced endoscopy, Neuroendoscope, Pituitary neoplasm

## Abstract

Precise tissue differentiation is vital in neurosurgery, especially during endoscopic endonasal surgery (ETS), where visual information is critical. The Yellow Enhance (YE) mode, a novel image-enhanced endoscopy technology, emphasizes yellow pigments to potentially improve tissue differentiation. This study retrospectively evaluated the efficacy of YE mode in five cases (two primary pituitary neuroendocrine tumors, one recurrent skull base-invasive pituitary neuroendocrine tumor, one pituitary apoplexy, and one recurrent craniopharyngioma) using the Olympus VISERA ELITE III endoscope. Eight experienced neurosurgeons reviewed surgical videos and provided 40 structured evaluations. Statistical analyses (Kruskal-Wallis and Mann-Whitney U tests) compared scores among cases. Gross or near-total resection was achieved in all cases without neurological complications. YE mode improved differentiation between normal pituitary tissue and tumors in 80% of cases, but was less effective in cases like pituitary apoplexy with degenerative changes. Across 40 evaluations, 68% rated YE mode as “useful” or “somewhat useful,” while 20% noted limited utility in complex cases, such as recurrent craniopharyngiomas. YE mode shows promise in enhancing visual differentiation during ETS, particularly for normal pituitary tissue, but its utility depends on tissue characteristics. Larger prospective studies are needed to validate these findings and explore broader applications in neurosurgery.

**Supplementary Information:**

The online version contains supplementary material available at 10.1007/s10143-025-03485-2.

## Clinical trial number

Not applicable.

## Introduction

In neurosurgery, which deals with delicate neural tissues, precise differentiation between tissues is crucial. This involves using preoperatively input information (anatomical knowledge, imaging findings, past surgical experiences) in a top-down manner and integrating it with information collected during surgery (visual information, tactile information, surgical navigation) in a bottom-up process. Among these, the most important and indispensable information is the visual information obtained in the operating field. Regardless of how perfect the other information is, a good surgery cannot be performed if visibility is poor. Conversely, even with limited other information, a surgery can be somewhat successful if the visual information is overwhelmingly superior.

In recent years, endoscopic endonasal surgery (ETS) has been established as a minimally invasive surgical option not only for pituitary neuroendocrine tumors (PitNETs) but also for ventral skull base tumors [2, 5–7]. One of the factors that has significantly promoted the development of this neuroendoscopy is the improvement in visual information due to advances in optical systems. High-resolution cameras for rigid endoscopes are now widely available nationwide, allowing for high-resolution visualization of tumors and surrounding normal tissues, which can be utilized for meticulous surgical procedures.

However, if the goal is perfect differentiation between tissues, high resolution alone has limitations. For example, normal pituitary tissue and PitNET tissue appear similar, and this similarity cannot be easily resolved with resolution alone. Experienced pituitary surgeons may be able to judge based on top-down auxiliary information such as experience, but this is not easy for most neurosurgeons. To address this challenge, multiple imaging techniques have been explored [12]. Narrow Band Imaging (NBI), for instance, uses specific wavelengths to emphasize capillary proliferation and mucosal micro-pattern changes associated with cancerous tissues [11, 13, 16]. 

The Yellow Enhance (YE) mode, equipped in Olympus’s latest endoscope (VISERA ELITE III [Olympus, Tokyo, Japan]), is a new technology that can emphasize tissues containing yellow pigments in the body through color gamut adjustment of the endoscope system. Originally developed to improve the differentiation accuracy between fatty tissue and other tissues in the field of abdominal surgery, similar effects are expected in other organ surgical fields. This report discusses the efficacy of YE mode in the field of neurosurgery based on personal usage experience.

## Materials & methods

This retrospective study examined the efficacy of the YE mode in five cases (two primary PitNETs, one recurrent skull base invasive PitNET, one pituitary apoplexy, and one recurrent craniopharyngioma) performed using the Olympus VISERA ELITE III in September 2023. This exploratory study aims to generate preliminary data for a novel technology. The surgeries were performed by a single surgeon with abundant experiences (HH), and the efficacy of the YE mode was retrospectively evaluated through a questionnaire by eight neurosurgery specialists, including the primary surgeon (HH), based on paired intraoperative video assessments comparing YE mode and normal light mode in each case. Since YE mode is an image-enhancement feature that can be toggled intraoperatively, all evaluations inherently involved direct comparisons with normal light mode rather than an isolated assessment of YE mode alone. Evaluators reviewed side-by-side video sequences, allowing for a structured comparison of the two visualization techniques in real time. Their level of ETS surgical experience was divided into four categories ([i] inexperienced - only observed/assistant, [ii] surgeons with experience in 50 or less cases, [iii] surgeons with experience in 51–100 cases, [iv] surgeons with experience in more than 100 cases). The evaluation items in the questionnaire are shown in Table 1. The original data is available on Harvard Dataverse (10.7910/DVN/2GLORB).

**Table 1:** The evaluation questionnaires

Question	Answer options
Q0. Level of experience as a primary surgeon of endoscopic transnasal surgery	1. Almost no experience as a primary surgeon
	2. Some (< 50) experiences as a primary surgeon
	3. Moderate (50–99) experiences as a primary surgeon
	4. Sufficient (> 100) experiences as a primary surgeon
Q1. Compared to normal light, is YE mode useful for distinguishing tumors from normal tissues?	1. Definitely less useful than the normal light
	2. Somewhat less useful than the normal light
	3. Similar to the normal light
	4. Somewhat more useful than the normal light
	5. Definitely more useful than the normal light
Q2. Was YE mode helpful in this case?	1. Almost useless throughout the case
	2. Not useful in many situations, but there were some situations where YE mode was useful
	3. Not all, but there were many situations where YE mode was useful
	4. Very useful throughout the case
Q3. Which tissues does YE mode particularly help distinguish from tumors?	(Open answer, multiple answers possible)

YE = yellow enhance

The details of our surgical techniques are described in previous articles [8–10]. Briefly, a 0-degree endoscope was predominantly used during ETS, while a 30-degree oblique-viewing endoscope was employed as an adjunct for visualizing lesions extending laterally and superiorly. For PitNET, the standard endoscopic transsphenoidal approach was utilized. Following the dural opening, an extracapsular dissection technique was employed. The tumor-gland interface was identified on the anterior surface, and dissection was carefully carried out along this interface using semi-sharp dissectors and ring curettes. Adequate tumor debulking was performed to facilitate its mobilization. For craniopharyngiomas, the standard endoscopic transplanum/tuberculum approach was adopted.

This study was approved by The Research Ethics Committee, Graduate School of Medicine and Faculty of Medicine, The University of Tokyo (IRB#2231). This study was performed in line with the principles of the Declaration of Helsinki. Written informed consent was obtained from all the patients. This manuscript underwent English language refinement using ChatGPT (OpenAI, San Francisco, CA, USA), an artificial intelligence-based language model, to enhance clarity and grammar. The authors reviewed and edited the manuscript thoroughly after the use of the artificial intelligence tool to ensure the final content reflected their original intent and scholarly rigor.

### Statistical analysis

Statistical analyses were performed to evaluate differences in the subjective evaluations of the YE mode across different surgical cases. A Kruskal-Wallis test was conducted to determine overall differences in the evaluations of the YE mode across five distinct cases. Following a statistically significant result from the Kruskal-Wallis test, pairwise comparisons using the Mann-Whitney U test were performed to identify specific differences between the cases. The significance threshold was set at *p* < 0.05. JMP Pro 18.0 (SAS, Cary, NC, USA) was used for the statistical analyses. This case series has been reported in line with the PROCESS Guideline [1]. 

## Results

**Fig. 1:**
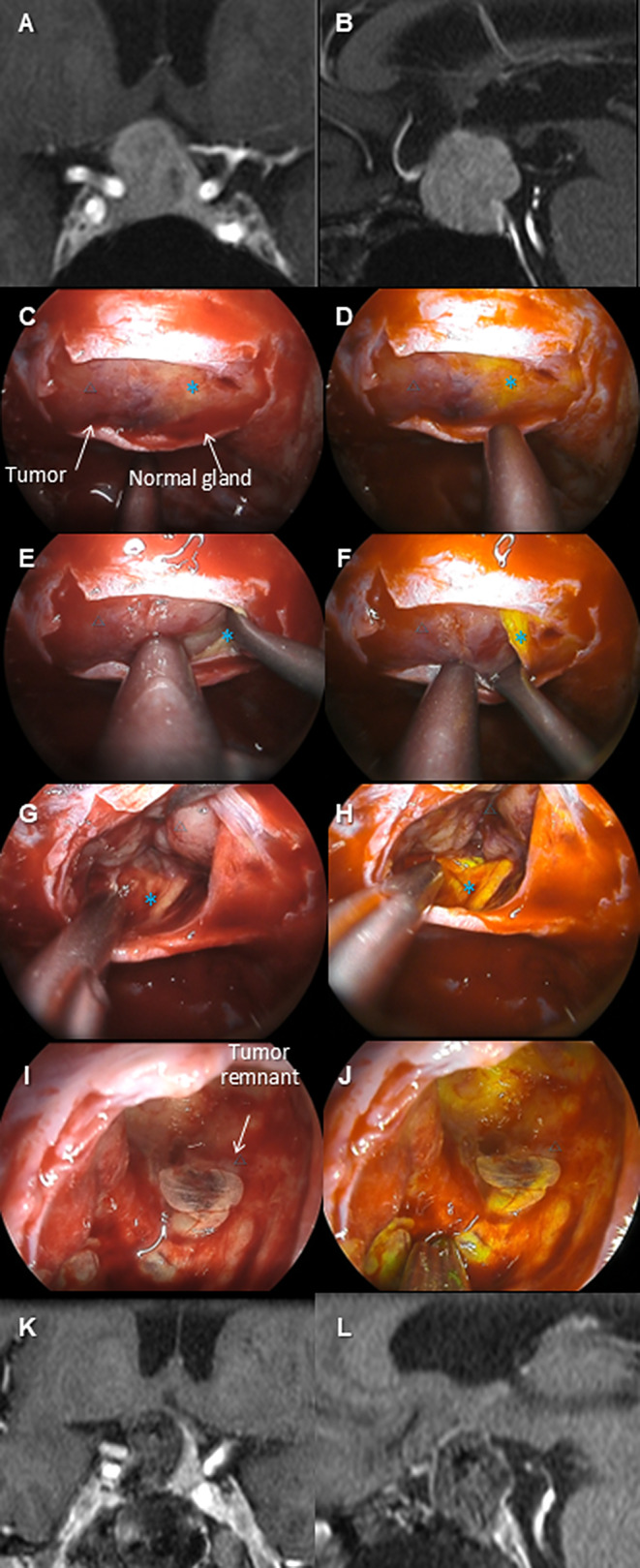
The preoperative MRIs (**A** and **B**, gadolinium-enhanced T1-weighted image), intraoperative pictures (**C**, **E**, **G**, and **I**, normal light; **D**, **F**, **H**, and **J**, Yellow Enhance [YE] mode), and postoperative MRIs (**I** and **J**, gadolinium-enhanced T1-weighted image) of case #2 are shown. This patient was a 73-year-old woman who presented with visual field disturbances accompanied by visual impairment due to pituitary neuroendocrine tumor. Preoperative MRI suggested that the normal pituitary gland was displaced to the left (**A**, **B**). During surgery, after wide exposure of the sellar region and dural incision, the tumor was exposed. Under YE mode observation, the yellow-tinted normal pituitary gland (*) was emphasized, enhancing the color contrast with the tumor (△), which facilitated easy differentiation (**C**–**H**). The tumor-pituitary boundary was dissected from the anterior side. The capsule became fragile toward the superior part, necessitating some intramembranous resection. The YE mode more clearly visualized a small tumor remnant than the normal light (**I**, **J**), and complete resection was ultimately achieved. The visual field disturbance improved postoperatively. The YE mode distinctly enhances the color contrast between the tumor and the normal gland

**Fig. 2:**
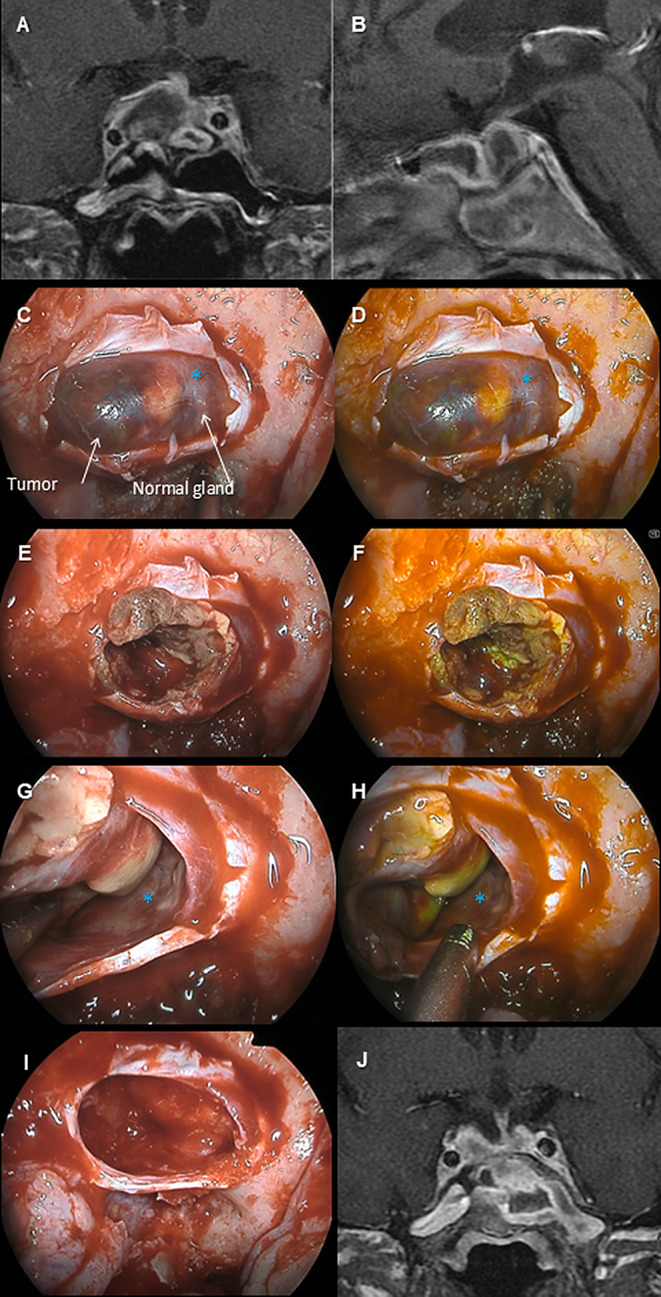
The preoperative MRIs (**A** and **B**, gadolinium-enhanced T1-weighted image), intraoperative pictures (**C**, **E**, **G**, and **I**, normal light; **D**, **F**, and **H**, Yellow Enhance [YE] mode), and postoperative MRI (J, gadolinium-enhanced T1-weighted image) of case #4 are shown. This patient is a 64-year-old man who developed pituitary apoplexy presenting with headache and hypopituitarism, with progressive oculomotor nerve palsy observed during the course, leading to surgery. Preoperative imaging suggested that the normal pituitary gland was displaced to the left (**A**, **B**). Intraoperative findings showed that the tumor tissue (△) had undergone yellowish degeneration due to apoplexy, and under YE mode, the tumor’s yellowish tint was more pronounced than the normal pituitary (*) (**C**–**H**). The degenerated tumor tissue was dissected and removed while switching between YE mode and normal light as needed, achieving extracapsular resection (**I**). His postoperative MRI showed no residual tumor (**J**), and the oculomotor nerve palsy gradually improved postoperatively, eventually resolving without sequelae. Overall, YE mode appears to enhance yellow tint, not only of the normal gland but also of the necrotic component of the tumor

**Fig. 3:**
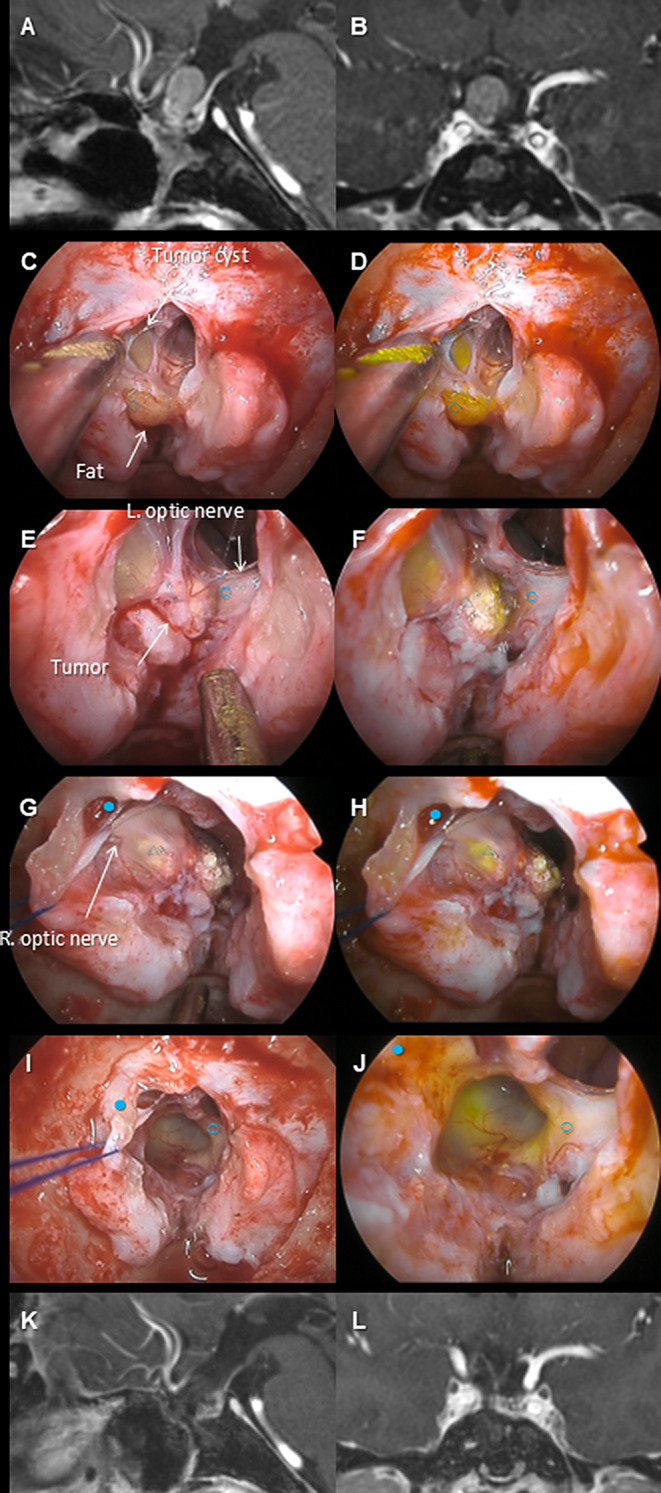
The preoperative MRIs (**A** and **B**, gadolinium-enhanced T1-weighted image), intraoperative pictures (**C**, **E**, **G**, and **I**, normal light; **D**, **F**, **H**, and **J**, Yellow Enhance [YE] mode), and postoperative MRIs (**K** and **L**, gadolinium-enhanced T1-weighted image) of case #5 are shown. This patient was a 50-year-old man with recurrent craniopharyngioma, previously operated on five times transnasally and twice via craniotomy, along with two radiation treatments since the age of 14, and presented with progressive visual field and visual acuity disturbances during imaging follow-up, leading to the diagnosis of tumor recurrence (**A**, **B**). The tumor was strongly compressing the left optic nerve and chiasm, and it was decided to resect it from below via ETS. The sellar region contained fat tissue (◇) from previous surgeries, which was strongly emphasized under YE mode. The tumor cyst and tumor (△) were pressing against the optic nerves (left, ○; right, ●), and under YE mode, the tumor tissues adjacent to the optic nerves was more emphasized, allowing for the creation of a boundary through sharp dissection (**C**–**H**). Ultimately, near-total resection was performed as visual-evoked potential decline was noted during the separation of the residual cyst wall from the optic chiasm (**I**, **J**). Postoperative imaging showed no apparent residue (**K**, **L**), and visual field and visual acuity disturbances improved to their original levels. Since both cystic and nodular components of the tumor are more strongly highlighted than the optic nerve with YE mode, the dissection plane between them is easily found

The summary of the cases is shown in Table 2. Complete or near-complete resection was achieved in all cases without neurological complications. The efficacy of the YE mode was distinctly recognized in some cases (e.g., Case #2), deemed less effective in others (e.g., Case #4), and mixed evaluations in yet others (e.g., Case #5). The details of three representative cases are shown (*Case #2*: Fig. 1A–F, Video 1; *Case #4*, Fig. 2A–F, Video 2; *Case #5*: Fig. 3A–F, Video 3).

**Table 2:** Summary of the patients

Case	Diagnosis	Primary vs. recurrent	Age, years	Sex	Symptom	Approach	EOR	Complication
#1	PitNET	Primary	71	M	Minor visual field deficit	Trans-sellar	GTR (extracapsular)	None
#2	PitNET	Primary	73	F	Vision loss	Trans-sellar	GTR (extracapsular)	None
#3	PitNET (clival & CS invasion)	Recurrent	43	F	Asymptomatic (progressive recurrence)	Trans-clival, trans-cavernous	GTR	None
#4	PitNET (apoplexy)	Primary	64	M	Headache, hypopituitarism, progressive oculomotor palsy	Trans-sellar	GTR (extracapsular)	None
#5	Craniopharyngioma	Recurrent	50	M	Vision loss	Trans-planum, trans-tubercular	NTR	None

CS = cavernous sinus; EOR = extent of resection; F = female; GTR = gross total resection; M = male; NTR = near total resection; PitNET = pituitary neuroendocrine tumor.

GTR indicates complete tumor resection without image-based evidence of tumor remnant, while NTR indicates > 99% tumor resection without image-based evidence of tumor remnant

### Questionnaire results

**Fig. 4:**
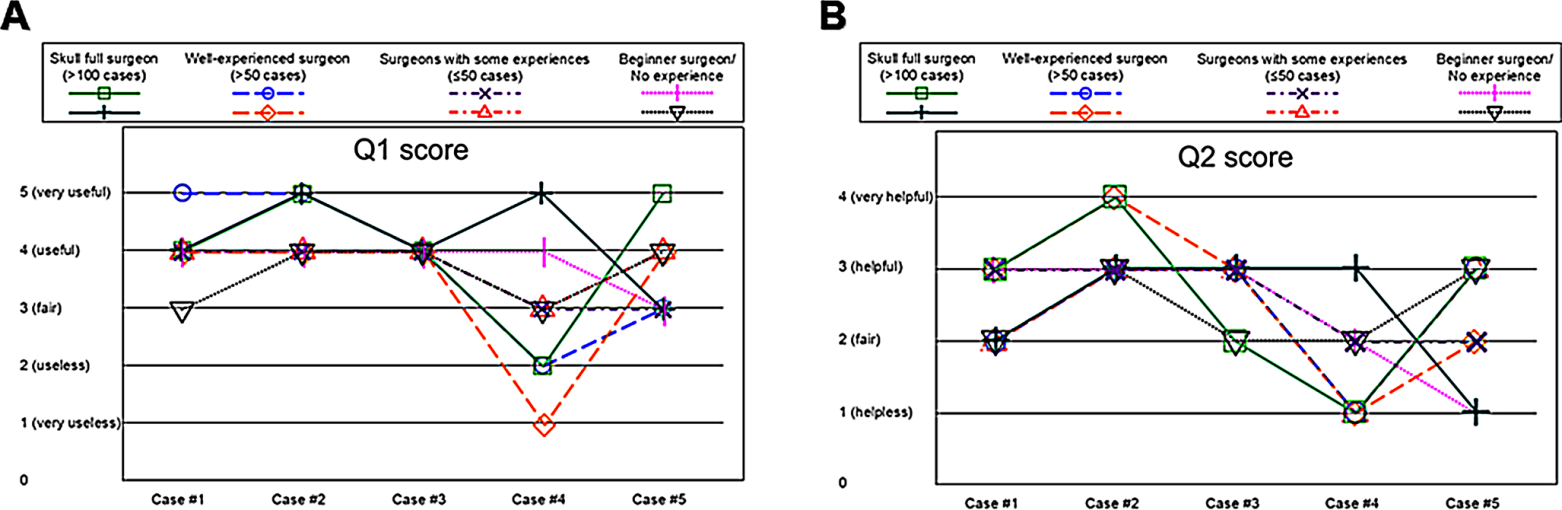
The distributions of Q1 (**A**) and Q2 (**B**) scores among evaluators across different cases are shown. Each colored line represents the evaluation of an individual evaluator

A total of 40 responses (5 cases × 8 evaluators) were obtained. Regarding the Q1 question ‘Is the YE mode useful for differentiating between tumor and normal tissue?’ (Fig. 4A), Case #4 received negative responses from 3 out of 8 evaluators (38%), stating that ‘normal light is certainly better’ or ‘normal light is somewhat better’, but no negative responses were noted in other cases. Regarding the Q2 question ‘Was the YE mode useful in the particular case?’ (Fig. 4B), 4 out of 8 evaluators (50%) in Case #4, and 1 evaluator (13%) in Case #5 rated it as ‘not useful’, while in other cases, most responses were positive, stating that the YE mode was ‘useful’ or ‘somewhat useful’ (Case #1: 6 evaluators, Case #2: 8 evaluators, Case #3: 6 evaluators, Case #4: 1 evaluator, Case #5: 3 evaluators).

The Kruskal-Wallis test revealed significant differences across the five cases for both Q1 (*p* = 0.0123) and Q2 (*p* = 0.0022), indicating variability in how the YE mode was perceived. Regarding the Q1, pairwise comparisons using the Mann-Whitney U test showed that the YE mode was rated significantly less useful in the pituitary apoplexy case (Case #4) compared to: primary PitNET (Case #1, *p* = 0.0406), recurrent PitNET with skull base invasion (Case #3, *p* = 0.0234), primary PitNET (Case #2, *p* = 0.0141). No significant differences were found between other cases, indicating similar usefulness in distinguishing tissues in primary and recurrent PitNET cases. Regarding the Q2, pairwise comparisons demonstrated that the YE mode was rated significantly less helpful for pituitary apoplexy (Case #4) compared to all other cases: primary PitNET (Case #1, *p* = 0.0045), recurrent PitNET (Case #3, *p* = 0.0045), primary PitNET (Case #2, *p* = 0.0069), recurrent craniopharyngioma (Case #5, *p* = 0.0392). No significant differences were observed between the non-apoplexy cases. Boxplots demonstrated a consistent trend, with pituitary apoplexy cases showing lower scores and greater variability.

The YE mode was particularly effective in distinguishing the pituitary gland from tumors, with 27 (68%) positive evaluations across all cases. The optic apparatus was identified as a structure that could be differentiated from tumors using the YE mode in six instances, with positive feedback in Cases #2 and #5. The mode also demonstrated some utility in identifying fibrous tissues, with four positive responses observed, primarily in Cases #2 and #5. Fat tissue and tumor cysts were only present in the surgical field of Case #5, where they were each noted by one evaluator as distinguishable using the YE mode.

## Discussion

The YE mode was effective in differentiating tissues in ETS. The normal pituitary, often showing a stronger yellow tint compared to PitNET, had its color contrast enhanced by YE mode, improving differentiation. Besides the normal pituitary, the optic nerve, fibrous tissues, tumor components of craniopharyngiomas, and adipose tissue in recurrent cases also showed improved differentiation thanks to the yellow tint enhancement. Importantly, however, the statistical analysis confirmed significant differences in the perceived efficacy of the YE mode across surgical cases. In particular, pituitary apoplexy cases were rated significantly lower in both usefulness for tissue differentiation and overall helpfulness, likely due to tissue degeneration leading to ambiguous color contrast. Conversely, the YE mode was perceived as consistently helpful for primary and recurrent PitNETs. These findings highlight the importance of case-specific considerations when applying image-enhanced endoscopy and emphasize the need for a flexible approach when interpreting visual cues provided by the YE mode.

In many cases, the influence of the surgeon’s experience level on the evaluation did not appear to be significant, but evaluations were somewhat mixed in Cases #4 and #5. This variation seemed to be more related to individual preferences rather than experience level. In Case #4, the tumor’s yellowish degeneration was more pronounced than the normal pituitary, while in Case #5, the tumor components, fat tissue from previous surgeries, and the optic nerve had varying degrees of yellow tint enhancement. Surgeons noted that it could be confusing to assume that the normal pituitary would always be emphasized in yellow, but the enhancement of yellow tint differences between tissues could still contribute to differentiation.

Even for tissues other than the pituitary, the yellow tint enhancement can improve differentiation. The optic nerve, for instance, was often found to benefit from this in PitNET and craniopharyngioma surgeries. In recurrent cases, differentiation from fibrous connective tissue may also improve. This suggests that YE mode could be effective in a variety of cases beyond initial or recurrent PitNETs.

Recently, several fluorophores (fluorescein, indocyanine green, 5-aminolevulinic acid) have become available, providing exogenous contrast through biochemical uptake and fluorescence emission [3, 4, 14, 15]. In contrast, YE mode enhances intrinsic tissue pigmentation via optical spectrum adjustments, requiring no perioperative administration or excitation light, allowing immediate intraoperative use. However, fluorophores offer dynamic contrast, particularly in vascular imaging and fluorescence-guided tumor resection. A combined approach may further enhance tissue differentiation, and future studies should evaluate whether integrating YE mode with fluorophores improves intraoperative visualization.

When interpreting the results of YE mode and this study, several limitations should be noted. First, tissue differentiation remains subjective, as YE mode only provides visual cues as with other image-enhanced endoscopy systems like NBI [11, 16]. Therefore, interpretation depends on the surgeon’s color perception and experience. This variability among neurosurgeons could impact the utility of YE mode. Second, intraoperative conditions, such as blood contamination or lighting variations, may affect color contrast, requiring careful fluid management and illumination adjustments. Third, certain tumor types with inherent yellow pigmentation, such as Crooke’s cell adenomas, may reduce the effectiveness of YE mode, limiting its applicability in specific cases. Furthermore, this study is retrospective and based on a small sample size (*n* = 5), which limits generalizability. While multi-expert evaluations helped mitigate bias, a prospective study with a larger cohort is necessary to validate these findings. Future research should also incorporate quantitative image analysis or reasonable color differentiation techniques to objectively compare YE mode with conventional visualization methods.

In summary, although the clinical advantages are to be determined, the YE mode has the potential to improve surgical outcomes in ETS by enhancing visual differentiation between tissues, especially the normal pituitary. Surgeons should remain flexible in their use of this mode and observe color differences without rigid assumptions.

## Conclusion

The YE mode represents a promising image-enhancement tool with the potential to improve surgical outcomes in endoscopic transnasal surgery (ETS) by enhancing visual differentiation between tissues, particularly the normal pituitary. Although the clinical advantages are yet to be fully determined, this mode can support more precise dissection and tissue identification. However, its effectiveness varies with tissue characteristics, and surgeons should remain flexible in interpreting color differences rather than relying rigidly on the color contrast. To validate these findings and generalize their clinical implications, larger, prospective studies are essential.

## Electronic supplementary material

Below is the link to the electronic supplementary material.

Supplementary Material 1

Supplementary Material 2

Supplementary Material 3

Supplementary Material 4

## Data Availability

The original data is available on Harvard Dataverse (https://doi.org/10.7910/DVN/2GLORB).

## Abbreviations

EOR: Extent of resection
GTR: Gross total resection
ETS: Endoscopic transnasal surgery
NBI: Narrow band imaging
NTR: Near-total resection
PitNET: Pituitary neuroendocrine tumor
YE: Yellow Enhance
