# Supplementary material for: Yellow enhance mode is useful for distinguishing tissues in endoscopic transnasal surgery: case series with preliminary results
Source: Neurosurg Rev. 2025 Apr 2;48(1):346. doi: 10.1007/s10143-025-03485-2 (PMC11965165; doi:10.1007/s10143-025-03485-2)
Supplement: Supplementary file 4 — Supplementary Material 4 [file 10143_2025_3485_MOESM4_ESM.docx]

**Video 1.** This video demonstrates an endoscopic transnasal surgery for a pituitary neuroendocrine tumor (Case #2). The Yellow Enhance (YE) mode highlights the color contrast between the tumor and the normal gland, facilitating the identification of the interface on the anterior surface. An extracapsular tumor resection is subsequently performed. Gross total resection was achieved.

**Video 2.** This video demonstrates an endoscopic transnasal surgery for a pituitary apoplexy (Case #4). In comparison to ordinary pituitary neuroendocrine tumor, the degenerated tumor has more yellow tints than the normal gland does. This could lead to some difficulties with distinguishing tumor tissue from the gland. Gross total resection was achieved.

**Video 3.** This video demonstrates an extended transsphenoidal exposure (expanding beyond the previous extent) for a recurrent craniopharyngioma. Immediately after durotomy, the yellow tint of the previously grafted fat is enhanced using the YE mode. The yellow tints of the tumor vary between portions but are largely helpful in distinguishing the tumor from the optic nerve, which shows less yellow. The remaining yellow tint on the optic chiasm suggests the presence of a thin layer of tumor. However, tumor removal must be halted due to a reduction in the VEP signal during adhesion dissection. Consequently, nearly total resection was achieved.
